# Comparison of the Structural Properties and Nutritional Fraction of Corn Starch Treated with Thermophilic GH13 and GH57 α-Glucan Branching Enzymes

**DOI:** 10.3390/foods8100452

**Published:** 2019-10-02

**Authors:** Inmyoung Park, Minjeong Park, Naeun Yoon, Jaeho Cha

**Affiliations:** 1 Department of Oriental Food and Culinary Arts, Youngsan University, Busan 48015, Korea; inmpark@ysu.ac.kr; 2 Department of Microbiology, College of Natural Sciences, Pusan National University, Busan 46241, Korea; 3 Microbiological Resource Research Institute, Pusan National University, Busan 46241, Korea

**Keywords:** starch, α-glucan branching enzyme (GBE), branching activity, in vitro digestibility

## Abstract

Two thermophilic 1,4-α-glucan branching enzymes (GBEs), *Cb*GBE from *Caldicellulosiruptor bescii* and *Ph*GBE from *Pyrococcus horikoshii*, which belong to the glycoside hydrolase family 13 and 57 respectively, were cloned and expressed in *Escherichia coli*. Two GBEs were identified to have α-1,6 branching activity against various substrates, but substrate specificity was distinct. Starch was modified by two GBEs and their in vitro digestibility and structural properties were investigated. Short-branched A chains with a degree of polymerization (DP) of 6–12 increased with *Cb*GBE-modified starch, increasing the proportion of slow digestible and resistant starch (RS) fractions. *Ph*GBE-modified starch resulted in an increase in the RS fraction only by a slight increase in part of A chains (DP, 6–9). Compared to the proportion of control not treated with GBE, the proportion of α-1,6 linkages in *Cb*GBE- and *Ph*GBE-modified starch increased by 3.1 and 1.6 times. ^13^C cross polarization/magic angle sample spinning (CP/MAS) NMR and XRD pattern analysis described that GBE-modified starches reconstructed double helices but not the crystalline structure. Taken together, *Cb*GBE and *Ph*GBE showed distinct branching activities, resulting in different α-1,6 branching ratios and chain length distribution, and double helices amount of starch, ultimately affecting starch digestibility. Therefore, these GBEs can be used to produce customized starches with controlled digestion rates.

## 1. Introduction

Starch is the most abundant and important polysaccharide consumed as an energy source by humans. Starch is divided into three categories based on its rate of digestion to glucose units in the small intestine: rapidly digestible starch (RDS), slowly digestible starch (SDS), and resistant starch (RS) [1]. SDS is slowly but completely digested in the small intestine, leading to a gradual increase in postprandial blood glucose levels. RS is not digested in the small intestine. It moves to the large intestine, where it is fermented by the intestinal microflora [2]. SDS and RS have low glycemic index (GI) values. The consumption of low-GI foods has health benefits that include a reduced risk of chronic metabolic diseases, such as diabetes, obesity, and cardiovascular disease [3].

Enzymatic modification is one of the preferred methods of producing modified starch with high SDS and RS contents. To reduce the enzymatic hydrolysis of starch and achieve low-GI values, enzymes like pullulanase, isoamylase, maltogenic amylase, α-glucanotransferase, amylosucrase, and glycogen branching enzyme have been applied either individually or in pairs. These enzymes catalyze the partial hydrolysis and debranching, or elongated amylose/amylopectin (AM/AP) chains, resulting in low-GI starch [4,5,6,7,8].

The 1,4-α-glucan branching enzyme (GBE, amylo-(1,4→1,6)-transglycosylase, EC 2.4.1.18) is a starch-modifying enzyme that catalyzes the formation of a new branching point with an α-1,6 glycosidic linkage after cleaving α-1,4 glycosidic linkages [9]. GBEs belong to either the glycoside hydrolase (GH) family 13 or 57. The GH57 family has been newly added based on two α-amylase sequences lacking the conserved sequence regions of GH13 enzymes [10]. Although they use the same catalytic mechanism [11], the substrate specificities of the GBEs belonging to these two families are also slightly different [12,13]. These different branching activities result in different side chain branch distribution and different molecular weight distribution [14]. Some studies on starch modification by GBEs have revealed that GBE-treated starches have an increased number of short branched chains (A or A and B_1_) and a decreased number of long branched chains (B_2_ and B_3_) and form highly branched α-glucan- and AP-like clusters [4,14,15,16]. The hydrolysis of α-1,6 linkages by the action of amylases in the gastrointestinal tract is a rate-limiting step. As a result, the presence of highly branched α-glucan may lead to slow starch digestion [4,14,15,16].

To improve the reaction activity between mesophilic enzymes and starch, starch-modifying enzymes are applied to solubilized starch after heating (gelatinization) and subsequent cooling to the optimal enzyme reaction temperature [17]. However, the use of thermophilic enzymes would be a cost-effective strategy for industrial applications as it saves much of the cooling time and prevents microbial contamination at the high temperatures used. Recently, we expressed a GBE in the GH57 family from hyperthermophilic archaeon *Pyrococcus horikoshii* OT3 (*Ph*GBE) in *Escherichia coli* and successfully solved a three-dimensional structure, allowing the determination of their branching activities and active sites [18].

In the present study, the enzymatic properties of two thermophilic enzymes, GH57 *Ph*GBE and GH13 *Cb*GBE from *Caldicellulosiruptor bescii*, were compared and modified starches were produced by using these two GBEs. In vitro digestibility of the GBE-modified starches was assessed and the structural properties that included branch points, side chain length distribution, and contents of double helices were explored. The findings clarified how these structural parameters are related to starch digestibility and identified the effects of the GBEs belonging to different family (GH13 vs. GH57).

## 2. Materials and Methods

### 2.1. Chemicals and Reagents

Maltooligosaccharides (degree of polymerization (DP), 1−7), AM (A-0512), and AP (A-8515) from potato starch; normal corn starch (S-4126), high-AM corn starch (A-7043), pancreatin (P-7545, activity 8 × USP/g), and amyloglucosidase from *Aspergillus niger* (A-7095; activity, ≥ 300 U/mL); and a glucose assay kit (GAGO20) were purchased from Sigma-Aldrich (St. Louis, USA). Isoamylase (Lot 130103a) from *Pseudomonas* sp. (activity, 280 U/mg) was obtained from Megazyme International Ltd. (Wicklow, Ireland). All other chemicals were of reagent grade and were purchased from Sigma-Aldrich.

### 2.2. Construction of PhGBE and CbGBE Overexpression Vectors

To detect GBE genes, PCR amplification of *ph_RS06575* (accession number WP_010885475) and *athe_RS02765* (accession number WP_015907139) was performed using *P. horikoshii* OT3 and *C. bescii* DSM6725 genomic DNA as templates, respectively. The *phGBE-*specific primers *Ph*GBE-F (5′-TTCATATGAAAGGATACCTAACGTTTGTTCTACA-3′) and *Ph*GBE-R (5′-AGCTCGAGAAGGGTTGATTTCTTCTTTTC-3′) containing *Nde*I and *Xho*I restriction enzyme sites (underlined) and the *CbGBE-*specific primers *Cb*GBE-F (5′-TGGAATTCATGATAAAAAAAGTAAAATCTAC-3′) and *Cb*GBE-R (5′-TCCTCGAGTATCTGAATATTGTTGTC-3′) containing *EcoR*I and *Xho*I sites were used. PCR was performed using 2.5 U of PrimeSTAR polymerase (Takara Bio, Otsu, Japan) under the following conditions: 25 cycles at 98 °C for 10 s (denaturation), 55 °C for 5 s (annealing), and 72 °C for 2 min (extension). The 1680-bp (*PhGBE*) and 1950-bp (*CbGBE*) PCR products were purified using a PCR purification kit (Elpis Biotech, Daejeon, Korea) and then digested using *Nde*I and *Xho*I or *EcoR*I and *Xho*I, respectively. The digested DNA fragments were separately ligated into the pET29b(+) vector (Novagen; Merck KGaA, Darmstadt, Germany), which was digested using the same restriction enzymes. The resulting expression vectors pET29b::PhGBE and pET29b::CbGBE encoded *Ph*GBE and *Cb*GBE with a C-terminal hexa-histidine tag, and its sequence was confirmed by DNA sequencing. The pET29b::PhGBE and pET29b::CbGBE were transformed into *E. coli* BL21(DE3), and the transformants were selected as described previously [18].

### 2.3. Expression and Purification of Recombinant PhGBE and CbGBE

The recombinant proteins were produced in this *E. coli* expression system and purified using Ni-NTA agarose (GE Healthcare, Little Chalfont, UK) [18]. Protein concentrations were quantified according to the Bradford method, using bovine serum albumin (Sigma-Aldrich, St. Louis, USA) as a standard [19]. The purity and molecular mass of the proteins were checked by 12% (*w*/*v*) SDS-PAGE using DokDo MARK (Elpis Biotech, Daejeon, Korea).

### 2.4. GBE Activity Assays

The iodine assay was used for measuring branching activity. The assay is based on monitoring the decrease in absorbance of the glucan–iodine complex resulting from amylose branching. The reaction mixture (50 µL) containing 0.1% (*w*/*v*) amylose in 50 mM sodium acetate buffer (pH 6.0) was incubated with 0.2 µg of the purified *Ph*GBE and 0.09 µg of the purified CbGBE separately at 60 °C for 15 min. The reaction was terminated by adding 50 µL of 1 M HCl and neutralized by adding 50 µL of 1 M NaOH. Aliquots of 100 µL were mixed with 900 µL of iodine solution (0.02% (*w*/*v*) I_2_ and 0.2% (*w*/*v*) KI), and absorbance was measured at 660 nm. One unit (U) of GBE was defined as the amount of enzyme that caused a decrease in absorbance of 0.01 optical density (OD) units per min under the assay conditions [20]. The optimal pH and temperature of two GBEs were also examined using same method as mentioned in above.

### 2.5. Substrate Specificities of the GBEs

A reaction mixture (50 µL) containing 0.1% (*w*/*v*) of each substrate (AM, AP, high-AM starch, and normal corn starch) in 50 mM sodium acetate buffer (pH 6.0) was incubated with 5 U of *Ph*GBE or 2 U of *Cb*GBE at 60 °C for 12 h. After adding 10 U isoamylase in 50 mM sodium acetate buffer (pH 4.0) to the reaction mixture in equal volume, the total reaction mixture (100 µL) was incubated at 40 °C for an additional 12 h, and the reaction was stopped by boiling for 10 min. After centrifugation (13,000 *g*, 10 min), the supernatant was spotted on a silica gel 60 F_254_ plate (Merck, Darmstadt, Germany). The plate was placed in a chamber equilibrated with a solvent mixture containing isopropyl alcohol/ethyl acetate/distilled water (3:1:1, *v*/*v*/*v*) and developed by dipping it into a solution containing 0.3% (*w*/*v*) naphthol and 5% (*v*/*v*) H_2_SO_4_, followed by baking at 120 °C for 10 min to identify the spots.

### 2.6. Determination of the Side Chain Length Distribution

To determine the action pattern of *Cb*GBE and *Ph*GBE on AM, 0.2% of AM (*w*/*v*) in 50 mM sodium acetate buffer (pH 6.0) was incubated with 5 U of *Ph*GBE or 2 U of *Cb*GBE at 60 °C for 20 h. The products were analyzed using a high-performance anion-exchange chromatography (HPAEC) column with a pulsed amperometric detector (Dionex, Sunnyvale, CA, USA). The detailed method was described previously [18]. The side chain length distribution of GBE-modified starches is described in the Supplementary Materials and Methods.

### 2.7. Preparation of GBE-Modified Starches

A reaction mixture containing normal corn starch (0.2 g starch, 2%, *w*/*v*) in 50 mM sodium acetate buffer (pH 6.0) containing dimethyl sulfoxide (10%, *v*/*v*) was boiled with stirring for 30 min. After cooling the mixture to 60 °C, *Ph*GBE or *Cb*GBE (2000 U and 5000 U) was added to the starch slurry and incubated at 60 °C for 20 h. The reaction was stopped by heating in boiling water for 30 min. After removing the insoluble materials by centrifugation, the solution was precipitated by adding three volumes of absolute ethanol. The enzyme-modified starch was precipitated by centrifugation (7000 *g,* 30 min) at 4 °C and washed four times with distilled water. The modified starch was freeze-dried and pulverized. Starch that was incubated under the same conditions without GBE treatment was used as a control.

### 2.8. Determination of the Linkage Ratios

The ratios of α-1,4 and α-1,6 linkages in the GBE-treated normal corn starch samples were determined using a 400 MHz ^1^H nuclear magnetic resonance (NMR) spectrometer (JeolJNM-LA400 with LFG; JEOL, Tokyo, Japan). Each sample (40 mg) was dissolved in 2 mL of deuterium oxide (D_2_O; 99.9% in D), vortexed for 5 min, and boiled for 30 min with occasional vortexing to avoid starch aggregation. These samples were freeze-dried again and re-dissolved in D_2_O (20 mg/mL) prior to ^1^H NMR analysis. The reference (δ = 0) used was 3-(trimethylsilyl)propionic-2,2,3,3-d4 acid, and ^1^H NMR spectra were collected at 80 °C.

### 2.9. Solid-State ^13^C NMR with Cross Polarization/Magic Angle Sample Spinning

High-resolution solid-state ^13^C NMR with cross polarization/magic angle sample spinning (CP/MAS NMR) experiments were conducted using an AVANCE 500 (Bruker, Billerica, MA, USA) NMR instrument equipped with CP/MAS accessories at a frequency of 100 MHz under high-power decoupling conditions. Approximately 200 mg of starch was packed using a 4 mm-diameter rotor. Samples were spun at a rate of 5 kHz, with a spectral width of 3.1 kHz, acquisition time of 35 ms, and 2.2 k time domain points at 25 °C. At least 4000 scans were accumulated for each spectrum. The data processing and resonance peak spectrum integration were performed using the TOPSPIN 1.3 software (Bruker BioSpin, Silberstreifen, Germany). The double helix-to-amorphous ratio was obtained as described previously [21].

### 2.10. X-ray Diffraction Patterns and Relative Crystallinity

X-ray diffraction (XRD) analysis was performed using an X-ray diffractometer (D8 ADVANCE with DAVINCI; Bruker, Karlsruhe, Germany) with a LYNXEYE XE detector (Bruker) at 40 kV, 40 mA, and a CuK_α_ radiation with a wavelength of two. The starch sample was scanned at a diffraction angle (2*θ*) ranging from 3° to 40° and a step time of 1 s. The relative crystallinity (*RC*) was calculated using the following equation, as previously described [22] using the TOPAS software (Bruker):

$$RC\;\left(\%\right)=\frac{A_{c}}{A_{a}+A_{c}}$$

where *A*_a_ is the area of the amorphous region and *A*_c_ is the area of the crystalline region.

### 2.11. Determination of the Starch Fractions based on Digestibility

The starch fractions were determined according to a previously described method [1] with slight modifications. Briefly, a freeze-dried starch sample (20 mg) was dissolved in 0.75 mL of a 100 mM sodium acetate buffer (pH 5.2) with a glass ball (5 mm-diameter) in a 2-mL microtube. The microtube was pre-incubated in a shaking incubator at 37 °C (200 g, 20 min). The prepared enzyme solution (0.75 mL) was added to each microtube and then incubated for 20 min or 240 min. After the time had elapsed the microtubes were removed and boiled for 10 min to terminate the enzyme reaction, centrifuged (1000 *g*, 10 min), and the glucose content released at 20 min (G20) and 240 min (G240) was determined using a glucose assay kit. The total glucose (TG) content was determined by gelatinizing the starch, cooling it, adding 7 M KOH, and treating it with 20 μL amyloglucosidase. Free glucose (FG) was determined from a gelatinized starch sample in an acetate buffer solution. The values for RDS, SDS, and RS were calculated using the following formulas: RDS = (G20 − FG) × 0.9; SDS = ((G240 − FG) − (G20 − FG)) × 0.9; and RS = (TG − FG) × 0.9 − (RDS + SDS).

### 2.12. Statistical Analysis

All experiments were performed at least three times. The data were reported as the means with standard deviation. Analysis of variance (ANOVA) was performed and differences in means of samples were analyzed using Duncan’s multiple range tests (*p* < 0.05) in the SAS software (version 9.3., SAS, Cary, NC, USA).

## 3. Results and Discussion

### 3.1. Purification of Recombinant PhGBE and CbGBE and Assessment of Their Branching Activity for Various Substrates

**Figure 1:**
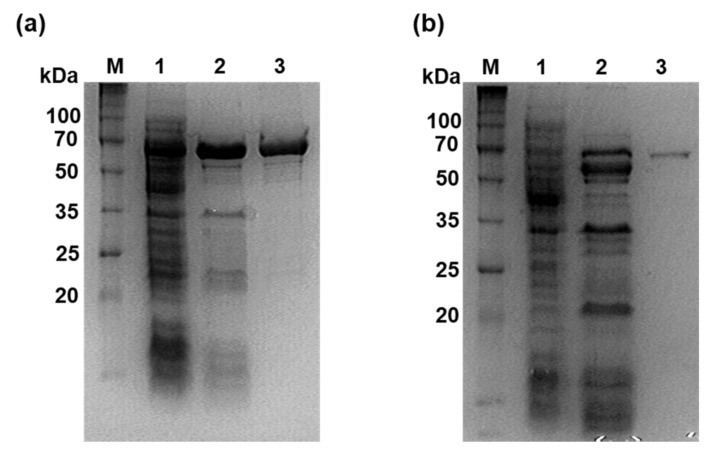
SDS-PAGE analysis of recombinant glucan branching enzyme from *Pyrococcus horikoshii* OT3 (*Ph*GBE) (**a**) and (*Caldicellulosiruptor bescii*) *Cb*GBE (**b**). Lane M, molecular size standards; lane 1, cell-free extracts; lane 2, proteins after heat treatment; lane 3, proteins after Ni-NTA affinity chromatography.

The *Ph*GBE from *P. horikoshii* OT3 and *Cb*GBE from *C. bescii* DSM 6725 were expressed in *E. coli* BL21(DE3) cells and purified by heat treatment followed by Ni-NTA chromatography. The *Ph*GBE and *Cb*GBE was purified 21.6- and 30.9-fold with a yield of 11.6% and 3.9%, respectively (Table S1). The molecular masses of the purified *Ph*GBE and *Cb*GBE, as estimated by SDS-PAGE, were approximately 66 kDa and 70 kDa, respectively (Figure 1).

Enzyme activity assays were performed at pH range 4−8 and temperature range 35−85 °C to determine the effect of pH and temperature of GBEs activity on AM as a substrate. The optimal pH and temperature for both GBEs was determined as 6.0 and 60 °C, respectively. *Cb*GBE showed higher activity than *Ph*GBE most experimented pH and temperature, however activity of *Cb*GBE significantly dropped over 70 °C (Figure S1). This overall result implies that both enzymes were successfully expressed as active forms in *E. coli* and have a thermostability.

**Figure 2:**
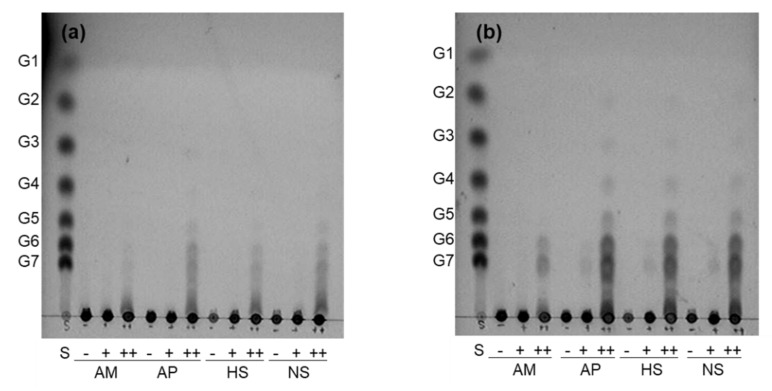
TLC analysis of the products obtained by reaction of *Ph*GBE (**a**) and *Cb*GBE (**b**) with various substrates, namely amylose (AM), amylopectin (AP), high-AM starch (HS), and normal corn starch (NS). Reaction mixtures were incubated at 60 °C with (+) or without (−) the enzyme. Isoamylase treatment (++) was performed after reaction with the enzyme. Lane S, standard maltooligosaccharide mixture containing glucose (G1), maltose (G2), maltotriose (G3), maltotetraose (G4), maltopentaose (G5), maltohexaose (G6), and maltoheptaose (G7).

The branching activity of GBEs with various substrates showed that high-molecular weight glucans containing both α-1,4 and α-1,6 glycosidic linkages, such as AP, normal starch (~75%−80% AP), and high-AM starch (~70% AM), were preferred, rather than AM, which contains only α-1,4 glycosidic linkages. *Cb*GBE showed a relatively stronger branching activity than *Ph*GBE, which is in agreement with a previous publication [14]. Thin-layer chromatography (TLC) revealed that *Cb*GBE effectively used AM and AP as substrates, while *Ph*GBE was less active for AM (Figure 2).

The reduced preference of the GH57 family for AM was previously observed for Tt-GBE in *Thermus thermophilus* HB8 and TkGBE in *Thermococcus kodakarensis* KOD1[12,13]. Thus, it was thought that GH57 GBEs prefer branched α-1,6 linked substrates, while GH13 GBEs do not differentiate between α-1,4 and α-1,6 linked substrates. *Cb*GBE was found to produce significant quantities of maltohexaose and maltoheptaose after isoamylase treatment through TLC analysis (Figure 2). However, since TLC has a detection limit for DP of <10, further studies are necessary to observe longer chain distributions.

### 3.2. Branch Activities of PhGBE and CbGBE on AM

**Figure 3:**
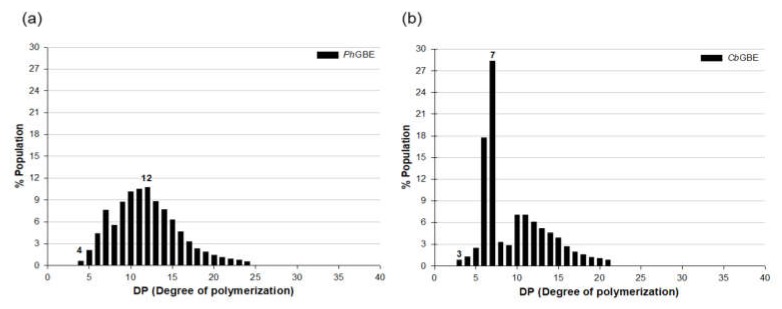
Chain length distribution of AM treated with *Ph*GBE (**a**) and *Cb*GBE (**b**) using by high-performance anion-exchange chromatography (HPAEC).

To overcome the TLC detection limits, HPAEC was used to extensively determine longer DP distribution using AM as a substrate. The chromatograms revealed variously sized glucan products (DP 4−24), suggesting that the two GBEs successfully interacted with AM (Figure 3).

*Cb*GBE predominantly produced side chains with DPs of six and seven, consistent with the TLC pattern (Figure 2). *Ph*GBE favored the production of side chains with DP 10–12 on AM; this branching pattern was significantly different between the two GBEs (Figure 3). This result suggested that *Cb*GBE generated a shorter branch chain than *Ph*GBE. However, previous research reports showed differences in the results of the branch activities of GH13 and GH57. Palomo et al. [13] reported that the thermophilic GBEs of the GH57 family principally produce shorter side chains (DP 6–7) compared with those produced by the GH13 family, which preferentially transfer chains with DP 10–14. Conversely, Sawada et al. [23] reported that the chain length profiles of reaction products of 10 different GBEs showed significant variation in terms of whether GBEs preferred A or B chains as their acceptor chains. Therefore, it is assumed that no correlation exists between the types of GBEs and branching properties.

### 3.3. Side Chain Length Distribution of GBE-Modified Starches

Normal corn starch was modified with *Ph*GBE or *Cb*GBE, and side chain length distribution was examined using HPAEC. In starches modified by the GBEs, the proportion of A chains increased, whereas those of the B_1_, B_2_, and B_3_ chains decreased (Table 1), which is consistent with the results of previous GBE studies [16,24].

Further, the branching activity of *Cb*GBE-treated starch was much more pronounced than that of *Ph*GBE; this was consistent with the TLC analysis results. After treatment with *Cb*GBE, the A chains (46.1%, DP 6−9 and 10−12) made up the largest fraction and a decrease in the proportions of the B_1_, B_2_, and B_3_ chains was observed (Table 1). However, the overall distribution of side chains in *Ph*GBE-modified starches was similar to that of control starch; the proportion of the shorter A chain (DP 6–9) increased slightly but that of the B_1_ chain was still predominant. The results of the detailed side chain length distribution on modified starches were dissimilar between the two GBEs. *Ph*GBE showed the greatest change in DP 6−9 from modified starch, but DP 10−12 was dominant when AM was used as a substrate, as shown in the previous result. The findings indicate that the branching activity of *Ph*GBE differs depending on the substrate.

### 3.4. Glycosidic Linkage Ratio of GBE-Modified Starches

^1^H NMR was used to analyze the ratio of α-1,6 linkages to the total glycosidic linkages in GBEs-modified starches. Native (normal corn starch), control, and two GBE-treated starches showed two distinct peaks at 5.4 and 5.0 ppm, indicating the chemical shift of anomeric protons in α-1,4 and α-1,6 linkages, respectively. The percentage of each linkage was calculated from the integrals of the corresponding peaks in the spectra [25]. The abundances of the α-1,6 linkage in the native and control starches were 2.8%, whereas the relative abundance of α-1,6 linkages in *Ph*GBE- and *Cb*GBE-treated starch was 4.4% and 8.6%, respectively (Table 2). The findings indicated that the branching ratio was significantly increased by 1.6 and 3.1-times in *Ph*GBE- and *Cb*GBE-treated starch, respectively, compared to that of the control, and that the branching ratio differed significantly (*p* < 0.05) between the two GBE-treated starches. Previous studies reported that α-1,6 linkages increased 7.1% and 8.2% after GBE treatment in waxy corn starch [4,16].

### 3.5. ^13^C CP/MAS NMR and XRD Analysis

**Figure 4:**
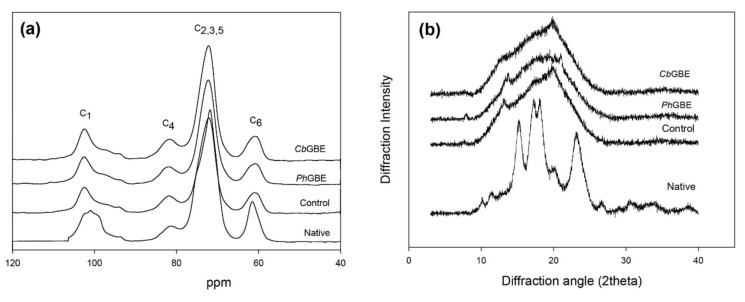
^13^C cross polarization/magic angle sample spinning (CP/MAS) NMR spectra (**a**) and XRD patterns (**b**) of native, control, *Ph*GBE, and *Cb*GBE-treated normal corn starches.

Starch crystallinity based on the ratio of double helices and amorphous (single-chain) sites, which is termed short-range molecular order, was determined by solid-state ^13^C CP/MAS NMR, whereas the long-range molecular order (double-chain) of crystallinity was determined by XRD. CP/MAS resonance peaks are specific for amorphous sites in the 80–87 ppm and 103–104 ppm regions, whereas signals at 99–102 ppm are specific for the double helices [6,21]. The integration of these peaks was used to obtain the percentage of double helices and amorphous sites of starch, as shown in Figure 4 and Table 3.

The double helix-to-amorphous site ratio for native corn starch was 42.1:57.9, consistent with that observed in a previous report [21]. Control starch showed a 23.3% double helix content. The double helix-to-amorphous site ratio was 30.4:69.6 for *Cb*GBE-treated starches and 29.1:70.9 for *Ph*GBE-treated starches. The degree of double helix of GBE-treated starches determined through ^13^C CP/MAS NMR analysis was lower than that of the native starch and higher than that of the control starch. ^13^C CP/MAS NMR examination also showed that the crystalline and amorphous regions of *Ph*GBE- and *Cb*GBE-treated starches did not differ significantly (*p* > 0.05).

Native normal corn starch displayed a typical A-type pattern through XRD analysis, with major diffraction peaks observed at 15°, 17°, 18°, and 23° (2*θ*), as reported previously [26]. The control starch did not exhibit any peaks, indicating a loss of crystallinity and presence of amorphous regions only (Figure 4). After treatment with the two different GBEs, calculation of relative crystallinity (*RC*) was not possible using the TOPAS software. This indicated that GBEs-treated starches were unable to rebuild a crystalline structure (Table 3). This result was in accordance with the loss of crystallinity observed in the XRD pattern of branching enzyme-modified sweet potato starch [27]. The longer chains, DP 25−36 and DP ≥ 37, favor the formation of double helices, connect to intra- and interclusters, and are important for the formation of semicrystalline structures of starch granules [28]. However, GBE-modified starches are not formed effectively in intra- and interclusters as they constitute new shorter α-1,4 glucans, rather than long chains, and lead to the formation of imperfect double helices that prevent a recrystalline starch structure [6,25].

XRD can detect the formation of regularly repeating double helices. However, it is incapable of doing so in an irregularly packed molecular starch structure. Thus, no peak was presently detected using XRD. Moreover, the structural aspect of GBE-treated starch side chains was the ability to build a double helix, but not crystals. The large difference between double helix content (^13^C NMR) and crystallinity (XRD) was indicative of incomplete crystallinity and irregularly packed structures as well (Table 3).

### 3.6. In Vitro Digestibility

GBE-modified starches had decreased RDS contents and increased low-GI fractions, which included the SDS and RS contents. These increased SDS and RS contents were more significant in *Cb*GBE-treated starch than in the *Ph*GBE-treated starch. *Cb*GBE-treated starch had an SDS content of 9.3% and RS content of 18.7%. *Ph*GBE-treated starch had an increased RS content of up to 14.5% and a slightly decreased SDS content compared with that of the native corn starch and control. The combined SDS and RS contents of *Ph*GBE- and *Cb*GBE-treated corn starch increased by 19.4% and 28.0%, respectively (Table 4).

The results concurred with those of previous studies, which showed that GBE-treated starch increases the proportion of short chains and α-1,6 branch points, and that the SDS and RS fractions increase in response to GBE [4,29,30]. Specifically, starch containing a high proportion of short chains (DP < 13) has a high SDS content, and highly branched modified starch has a lower digestion rate due to slower hydrolysis of the α-1,6 linkages compared with that of α-1,4 linkages, which has been confirmed by in vivo, in vitro, and enzyme kinetic studies [29,31].

*Cb*GBE and *Ph*GBE-treated starches also exhibited an increase in short chains (A chains) and α-1,6-branch points, which were dependent on the nature of the GBE; the relative ratio of A chains and 1,6-branching points in *Cb*GBE-treated starches was much higher than that in *Ph*GBE-treated starches. *Cb*GBE transferred relatively large quantities of shorter A chains (DP 6−9, 25%), as well as overall A chains (DP 6−12, 46.1%), to the acceptor chains to form branch points, whereas *Ph*GBE formed slightly higher quantities of shorter A chains with DP 6–9, while longer A chains with DP 10–12 were formed in quantities similar to those of the control. *Cb*GBE increased the SDS and RS contents, while *Ph*GBE increased the RS content only. Therefore, we propose that the quantity of A chains and the DP distribution (DP 6−9 and DP 10−12) favors both RS and SDS formation (from *Cb*GBE), whereas the quantity of shorter A chains (DP 6−9) is related to RS formation (from *Ph*GBE). This suggests that A chains with DP 10–12 are related to the SDS content. This study also confirmed that a significant quantity of A chains (DP 6−12) and an increased number of branch points favor the formation of both SDS and RS fractions, slow the enzyme accessibility to starch and reduce the digestibility of starch.

## 4. Conclusions

Two thermophilic enzymes belonging to two different families, *Cb*GBE (GH13) and *Ph*GBE (GH57), showed different branching activities with the substrate they used. The branching activity of *Cb*GBE contained both α-1,4 and α-1,6 linkages, whereas *Ph*GBE showed limited activity for substrates containing α-1,4 linkages such as AM. Modified starch with GBEs showed *Ph*GBE was remarkable with DP 6−9 and *Cb*GBE was with DP 6−12 side chains. Subsequently, two types of GBE produced a different side chain distribution, degree of branch points, and nutritional fractions in starch. The *Cb*GBE increased the amount of SDS and RS, but *Ph*GBE only increased the amount of RS. The collective findings of this study indicate that GBEs could be effective for producing customized modified starch and control glucogenesis in vivo. Further, thermostable GBEs might be useful as a cost-effective strategy to reduce the processing time for the industrial production of starches with a low-GI value.

## Figures and Tables

**Table 1:** The side chain length distributions and α-1,6 linkages of GBE-treated corn starch.

Starch	Relative Peak Area (%)				
	DP 6−9 (A Chains)	DP 10−12 (A Chains)	DP 13−24 (B_1_ Chains)	DP 25−36 (B_2_ Chains)	≥DP 37 (B_3_ and Longer)
control ^1^	8.1 ± 0.2 ^c2,3^	14.7 ± 0.4 ^b^	56.0 ± 0.7 ^a^	15.9 ± 0.7 ^a^	5.1 ± 0.1 ^a^
*Ph*GBE	10.0 ± 0.4 ^b^	14.9 ± 0.2 ^b^	54.3 ± 0.4 ^b^	15.3 ± 0.6 ^a^	4.9 ± 0.5 ^a^
*Cb*GBE	25.2 ± 0.5 ^a^	20.9 ± 0.1 ^a^	42.1 ± 1.1 ^c^	7.8 ± 1.1 ^b^	2.1 ± 0.7 ^b^

^1^ Control means the starch that was incubated under the same conditions without GBE treatment. ^2^ Data are expressed as mean value and standard deviation. ^3^ Values with different superscript letters in each column are found to be significantly different (*p* < 0.05) by Duncan’s multiple range test.

**Table 2:** Relative abundance of α-1,4 and α-1,6 linkages in GBE-modified normal corn starches.

Starches	α-1,4 Linkage (%)	α-1,6 Linkage (%)
Native	97.2 ± 0.1 ^a1,2^	2.8 ± 0.1 ^c^
Control	97.2 ± 0.1 ^a^	2.8 ± 0.1 ^c^
*Ph*GBE-treated	95.6 ± 0.1 ^b^	4.4 ± 0.1 ^b^
*Cb*GBE-treated	91.4 ± 0.1 ^c^	8.6 ± 0.1 ^a^

^1^ Data are expressed as mean value and standard deviation. ^2^ Values with different superscript letters in each column are found to be significantly different (*p* < 0.05) by Duncan’s multiple range test.

**Table 3:** Crystallinity and molecular order of GBE-modified normal corn starches.

Starches	XRD Crystallinity (%)	^13^C CP/MAS NMR		Double Helix (NMR)–Crystallinity (XRD)
		Double Helix (%)	Amorphous (%)	
Native	42.9 ± 0.1 ^1^	42.1 ± 0.1 ^a^^3^	57.9 ± 0.0 ^c^	N/A
Control	N/A ^2^	23.3 ± 0.0 ^d^	76.7 ± 0.1 ^a^	23.3
*Ph*GBE-treated	N/A	29.1 ± 0.1 ^bc^	70.9 ± 0.1 ^b^	29.1
*Cb*GBE-treated	N/A	30.4 ± 0.0 ^b^	69.6 ± 0.0 ^b^	30.4

^1^ Data are expressed as mean value and standard deviation. ^2^ N/A, not applicable ^3^ Values with different superscript letters in each column are found to be significantly different (*p* < 0.05) by Duncan’s multiple range test.

**Table 4:** Nutritional starch fractions of GBE-modified normal corn starch.

Starches	RDS (%)	SDS (%)	RS (%)	SDS + RS
Native	85.3 ± 0.9 ^a1,2^	5.3 ± 1.8 ^c^	9.4 ± 1.5 ^d^	14.7
Control	83.8 ± 1.9 ^a^	5.7 ± 1.5 ^c^	10.5 ± 2.1 ^c^	16.2
*Ph*GBE-treated	80.6 ± 1.0 ^b^	4.9 ± 1.5 ^b^	14.5 ± 1.1 ^b^	19.4
*Cb*GBE-treated	72.1 ± 3.4 ^c^	9.3 ± 2.8 ^a^	18.7 ± 1.6 ^a^	28.0

^1^ Data are expressed as mean value and standard deviation. ^2^ Value with different superscript letters in each column are found to be significantly different (*p* < 0.05) by Duncan’s multiple range test.

## Supplementary Materials

The following are available online at https://www.mdpi.com/2304-8158/8/10/452/s1, Materials and Methods: Analysis of chain length distribution, Figure S1: pH (**a**) and temperature (**b**) on the activity of *Ph*GBE (closed symbol) and *Cb*GBE (open symbol), Table S1: Purification step of recombinant *Ph*GBE and *Cb*GBE.
