# Supplementary material for: Comparison of the Structural Properties and Nutritional Fraction of Corn Starch Treated with Thermophilic GH13 and GH57 α-Glucan Branching Enzymes
Source: Foods. 2019 Oct 2;8(10):452. doi: 10.3390/foods8100452 (PMC6835866; doi:10.3390/foods8100452)
Supplement: Supplementary file 1 [file foods-08-00452-s001.pdf]

Supplementary Materials and Methods

Analysis of Chain Length Distribution

The reaction mixture (1 mL) containing 0.2% (w/v) AM in 50 mM sodium acetate buffer (pH 6.0) was incubated with 50 μg of purified *Ph*GBE enzyme at 60°C for 20 h. The reaction was terminated by addition of 1 mL ethanol. The precipitate was obtained by centrifugation at 13,000*g* for 15 min and dried using a rotary vacuum evaporator. The dried pellet was resuspended in 10% (w/v) dimethyl sulfoxide (DMSO) and 50 mM sodium acetate (pH 4.0) and then incubated with 10 U of isoamylase at 40°C for 72 h. After the reaction was stopped by boiling, the supernatant obtained by centrifugation at 13,000*g* for 5 min was filtered through a 0.2 μm membrane filter and injected into a high performance anion exchange chromatography with a pulsed amperometric detector (HPAEC/PAD system, Dionex, CA, USA). A CarboPac™ PA-1 anion-exchange column (4 × 250 mm; Dionex) and a guard column were usede to separate the debranched samples. After the column was equilibrated with 150 mM NaOH, the filtered sample (50 μL) was eluted with multiple gradients of 600 mM sodium acetate in 150 mM NaOH at a flow rate of 1 mL/min. The linear sodium acetate gradient was as follows: 10–30% for 0–10 min, 30–40% for 10–16 min, 40–50% for 16–30 min, 50–60% for 30–52 min, 60–100% for 52–82 min. A mixture of maltooligosaccharides containing from glucose to maltoheptaose (DP 1–7, 0.005 mg/mL) was used as a standard. To ensure the hydrolytic activity of *Ph*GBE, the reaction mixture (1 mL) containing 0.2% AM in 50 mM sodium acetate buffer (pH 6.0) was incubated with 50 μg of *Ph*GBE at 60°C for 20 h. The reaction was terminated by boiling, and the supernatant obtained by centrifugation at 13,000*g* for 5 min was filtered by a 0.2 μm membrane filter and injected into a HPAEC/PAD system.

Table S1. Purification step of recombinant *Ph*GBE and *Cb*GBE

	Total activity (U)[1]	Total protein (mg)	Specific activity (U/mg)	Yield (%)	Purification fold
*Ph*GBE					
Cell-free extract	1253	14.85	84	100	1.0
Heat treatment	1088	0.66	1648	86.8	19.6
Ni-NTA	14.5	0.08	1817	11.6	21.6
*Cb*GBE					
Cell-free extract	2366	15.89	149	100	1.0
Heat treatment	1943	1.98	981	82.1	6.6
Ni-NTA	92	0.02	4607	3.9	30.9

[1]The activity unit (U) is the amount of enzyme that decreases the absorbance by 0.01 per min in under the assay conditions.

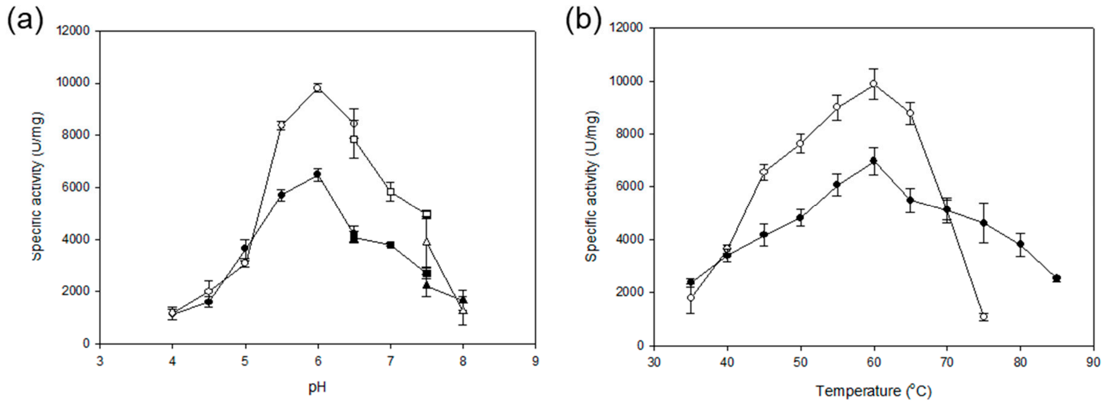

Figure S1. Effects of pH (a) and temperature (b) on the activity of *Ph*GBE (closed symbol) and *Cb*GBE (open symbol). The following buffers were used for optimal pH: sodium acetate (circle), Mops (square), and Tris-HCl (triangle). Error bars indicate standard deviations from three independent experiments.
